# 紫杉醇脂质体联合顺铂方案一线治疗晚期非小细胞肺癌的临床随机对照研究

**DOI:** 10.3779/j.issn.1009-3419.2012.04.03

**Published:** 2012-04-20

**Authors:** 新杰 杨, 卉 张, 靖颖 农, 敬慧 王, 曦 李, 权 张, 群慧 王, 远 高, 树才 张

**Affiliations:** 101149 北京，首都医科大学附属北京胸科医院肿瘤内科 Department of Medical Oncology, Beijing Chest Hospital, Capital Medical University, Beijing 101149, China

**Keywords:** 脂质体, 紫杉醇, 肺肿瘤, 临床研究, 化疗, Liposome, Paclitaxel, Lung neoplasms, Clinical trial, Chemotherapy

## Abstract

**背景与目的:**

紫杉醇联合顺铂方案（pacilitaxel plus cisplatin, TP）是目前一线治疗晚期非小细胞肺癌（non-small cell lung cancer, NSCLC）的标准方案之一。本研究旨在比较紫杉醇脂质体联合顺铂（liposome pacilitaxel plus cisplatin, LP）方案与TP方案一线治疗晚期NSCLC的近期疗效、远期生存及毒副反应。

**方法:**

100例患者随机分为两组，分别静脉注射紫杉醇脂质体和紫杉醇注射液150 mg/m^2^，第1天，联合顺铂75mg/m^2^，第1天-2天，21天一个周期。

**结果:**

100例患者均可评价疗效，其中LP组中位无进展生存期（progression free survival, PFS）为5.1个月，中位总生存期（overall survival, OS）为9.0个月，客观反应率（response rate, RR）为26%；TP组中位PFS为4.2个月，中位OS为9.3个月，RR为24%；两组比较均无统计学差异（*P*=0.110; *P*=0.342; *P*=0.890）。两组Ⅲ度+Ⅳ度毒性反应均无统计学差异（*P* > 0.05），LP组末梢神经炎发生率低于TP组（8% vs 28%, *P*=0.030）。

**结论:**

LP方案一线治疗晚期NSCLC疗效与TP方案相当，末梢神经炎发生率低于TP方案。

## Abstract (en)

**Background and objective:**

Paclitaxel plus cisplatin (TP) is used as the standard regimen for patients with advanced non-small cell lung cancer (NSCLC). In this study, we compared the response rate (RR), overall survival (OS), and toxicity of the combined chemotherapy regimen of liposomal paclitaxel plus cisplatin (LP) with those of TP as first-line treatments for advanced NSCLC.

**Methods:**

A total of 100 patients were randomly selected to be treated with liposomal paclitaxel or paclitaxel at a dose of 150 mg/m^2^ on day 1 plus cisplatin at a dose of 75 mg/m^2^ on days 1 and 2 per cycle every 21 days.

**Results:**

All 100 patients were eligible. The median progression free survival was 5.1 months *vs* 4.2 months, the median OS was 9.0 months *vs* 9.3 months, and RR was 26% *vs* 24% in the LP and TP arms, respectively. No significant difference was observed (*P*=0.110, 0.342 and 0.890, respectively). There was no significant difference between grades 3 and 4 toxicity of the arms (*P* > 0.05). Peripheral neuritis observed in the LP arm was significantly lower than that in the TP arm (8% *vs* 28%), and the difference was statistically significant (*P*=0.030).

**Conclusion:**

The effects of LP and TP as first-line therapies for NSCLC are similar. However, peripheral neuritis in the LP arm is significantly lower than in the TP arm.

紫杉醇是近十年来最有效的广谱抗肿瘤药物之一，治疗晚期非小细胞肺癌(non-small cell lung cancer, NSCLC)有效率为20%-42%。但由于其水溶性差，其聚氧乙烯蓖麻油与无水乙醇混合溶媒在体内降解易引起过敏反应并加重外周神经毒性，临床应用受到极大的限制。目前国内已经有多篇文献^[1-3]^报道紫杉醇脂质体较传统紫杉醇降低了药物的毒副作用，提高了临床耐受性，但有关紫杉醇脂质体是否也能给晚期NSCLC患者带来生存获益的相关文献却报道甚少。为比较紫杉醇脂质体(南京思科药业有限公司生产)联合顺铂(liposome paclitaxel plus cisplatin, LP)方案与紫杉醇注射液联合顺铂方案(paclitaxel plus cisplatin, TP)一线治疗晚期NSCLC的近期疗效、远期生存及毒副反应，首都医科大学附属北京胸科医院肿瘤内科从2007年5月-2009年3月对100例NSCLC患者随机给予LP或TP方案治疗，现将研究结果总结如下。

## 1 资料与方法

### 1.1 临床资料

入选标准：经细胞学和/或组织学确诊为NSCLC的Ⅲb期/Ⅳ期患者，既往未经过放化治疗，有符合RECIST标准的可测量病灶，年龄18岁以上，体能状况评分(performance status, PS)评分0分-2分，预计生存期 > 3个月，肝肾功能、心电图及血常规均未见明显异常，无症状的脑转移患者，依从性好。排除标准：妊娠期或哺乳期妇女，有症状的脑转移，无法控制的胸腔积液，存在其它肿瘤病史。采用随机分组软件开放式入组，预计入组例数每组不少于30例。最终入组LP组50例，包括男性34例，女性16例，年龄25岁-73岁，中位年龄53.2岁，Ⅲb期6例，Ⅳ期44例，PS 0分-1分45例，PS 2分5例，腺癌31例，鳞癌16例，腺鳞癌3例；TP组50例，包括男性32例，女性18例，年龄34岁-76岁，中位年龄55.2岁，Ⅲb期11例，Ⅳ期39例，PS 0分-1分44例，PS 2分6例，腺癌27例，鳞癌19例，腺鳞癌4例。两组患者临床资料相比差异无统计学意义(*P* > 0.05)，具有可比性(表 1)。

**1:** 两组患者临床资料 Clinical characteristics of the patients

Item	*n*	LP (*n*=50)Proportion (%)	*n*	TP (*n*=50)Proportion (%)	*P*
Sex					0.673
Male	34	68	32	64	
Female	16	32	18	36	
Age (year)					0.879
Median	53.2	-	55.2	-	
Range	25-73	-	34-76	-	
Stage					0.183
Ⅲb	6	12	11	22	
Ⅳ	44	88	39	78	
ECOG PS					0.749
0-1	45	90	44	88	
2	5	10	6	12	
Histology					0.713
Adenocarcinoma	31	62	27	54	
Squamous	16	32	19	38	
Squamous+adenocarinoma	3	6	4	8	
Cycles					0.988
Median	3.4	-	2.9	-	
Range	2-6	-	2-6	-	
TP: Paclitaxel plus cisplatin; LP: liposome paclitaxel plus cisplatin.					

### 1.2 治疗方案

LP组：紫杉醇脂质体150 mg/m^2^，第1天，静脉滴注3 h-4 h，顺铂75 mg/m^2^，第1、2天分2天静脉滴注，21天一个周期；TP组：紫杉醇150 mg/m^2^，第1天，静脉滴注3 h-4 h，顺铂75 mg/m^2^，第1、2天分2天静脉滴注，21天一个周期。两组化疗前均给予预防性抗过敏药物：LP组在使用紫杉醇脂质体前30 min给予地塞米松5 mg静注；TP组在使用紫杉醇前12 h、6 h分别给予地塞米松20 mg口服。两组均在化疗前30 min给予苯海拉明40 mg肌注，西咪替丁400 mg静注；化疗前后常规给予格拉司琼3 mg静注止吐治疗。化疗用药期间常规监测血压及心电。出现Ⅱ度以上的血液毒性给予升血治疗。每2个周期评价疗效及不良反应，化疗最多不超过6个周期，观察疗效直至疾病进展，随访生存情况。两组患者治疗周期差异无统计学意义(*P*=0.988)。

### 1.3 评价标准

主要研究终点为无进展生存期(progression free survival, PFS)，次要研究终点为总生存(overall survival, OS)、客观反应率(response rate, RR)和毒副反应。近期疗效按照RECIST实体瘤疗效标准，分为完全缓解(complete remission, CR)、部分缓解(partial remission, PR)、稳定(stable disease, SD)和进展(progressive disease, PD)，CR+PR为有效。PFS指从化疗开始至疾病进展的时间；OS指从化疗开始至死亡或末次随访时间。末次随访日期为2011年8月底，中位随访时间为20个月。毒副反应按NCI-CTC 3.0抗肿瘤药物毒性反应分级标准分为Ⅰ级-Ⅴ级。

### 1.4 统计学方法

采用SPSS 16.0统计软件进行分析。组间差异比较采用*χ*^2^检验、*Fisher's*精确检验法或秩和检验；生存资料分析采用*Kaplan-Meier*法，各因素比较采用*Log-rank*分析。*P* < 0.05为差异有统计学意义。

## 2 结果

### 2.1 近期疗效

见表 2。100例患者均完成两个周期以上化疗，疗效可评价。LP组50例，其中CR 0(0/50)，PR 26%(13/50)，RR 26%(13/50)；TP组50例，其中CR 0(0/50)，PR 24%(12/50)，RR 24%(12/50)，两组客观反应率相比差异无统计学意义(*P*=0.890)。

**2:** LP组与TP组疗效比较 Response and survival to treatment between LP arm and TP arm

Item	LP (*n*=50)	TP (*n*=50)	*P*
Response			0.890
CR	0 (0)	0 (0)	
PR	13 (26%)	12 (24%)	
SD	25 (50%)	24 (48%)	
PD	12 (24%)	14 (28%)	
CR+PR	13 (26%)	12 (24%)	
Survival			
Median PFS (month)	5.1	4.2	0.110
Median OS (month)	9.0	9.3	0.342
CR: complete remission; PR: partial remission; SD: stable disease; PD: progressive disease; PFS: progression free survival; OS: overall survival.			

### 2.2 毒副反应

两组主要毒副反应均为骨髓抑制、胃肠道反应和脱发，其中血液学毒性主要为贫血、白细胞下降及血小板减少，但两组的Ⅲ度+Ⅳ度毒性反应相比均无统计学差异(*P* > 0.05)。非血液学毒性反应中最常见为恶心呕吐，两组比较无统计学差异(*P* > 0.05)。LP组末梢神经炎发生率低于TP组(8% *vs* 28%)，差异具有统计学意义(*P*=0.030)。两组均无治疗相关性死亡(表 3)。

**3:** LP组与TP组治疗毒副反应比较 Toxic effects between LP arm and TP arm

Toxic effects	LP (*n*=50)	TP (*n*=50)	*P*
Anemia			0.084
Ⅰ-Ⅱ	17 (34%)	8 (16%)	
Ⅲ-Ⅳ	2 (4%)	1 (2%)	
Leucopenia			0.366
Ⅰ-Ⅱ	22 (44%)	25 (50%)	
Ⅲ-Ⅳ	25 (50%)	19 (38%)	
Thrombocytopenia			0.269
Ⅰ-Ⅱ	14 (28%)	8 (16%)	
Ⅲ-Ⅳ	2 (4%)	1 (2%)	
Nausea and vomiting			0.793
Ⅰ-Ⅱ	31 (62%)	28 (56%)	
Ⅲ-Ⅳ	6 (12%)	6 (12%)	
Perpheral neuritis			0.030
Ⅰ-Ⅱ	4 (8%)	13 (26%)	
Ⅲ-Ⅳ	0	1 (2%)	
Myositis			0.671
Ⅰ-Ⅱ	15 (30%)	18 (36%)	
Ⅲ-Ⅳ	0	0	
Allergic reaction			-
Ⅰ-Ⅱ	0	0	
Ⅲ-Ⅳ	0	0	
Baldness			0.271
Ⅰ-Ⅱ	39 (78%)	38 (76%)	
Ⅲ-Ⅳ	2 (4%)	6 (12%)	
Hepaic			> 0.999
Ⅰ-Ⅱ	3 (6%)	3 (6%)	
Ⅲ-Ⅳ	0	0	
Renal			> 0.999
Ⅰ-Ⅱ	2 (4%)	2 (4%)	
Ⅲ-Ⅳ	0	0	

### 2.3 远期生存

100例患者均观察至疾病进展，其中9例患者失访，其中LP组4例，TP组5例。LP组中位PFS为5.1个月，中位OS为9.0个月；TP组中位PFS为4.2个月，中位OS为9.3个月。两组PFS(图 1A)及OS(图 1B)相比均无统计学差异(*P*=0.110, *P*=0.342)。

**1:**
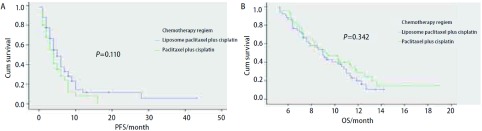
LP组和TP组PFS（A）和OS（B）比较 *Kaplan-Meier* estimates by treatment arm. Liposome paclitaxel plus cisplatin versus paclitaxel plus cisplatin. A: PFS; B: OS.

## 3 讨论

紫杉醇是从红豆杉的树皮中提取和纯化的具有紫杉烷环的化合物，是一种新型的抗肿瘤药物。主要通过与细胞微管结合，抑制其解聚，阻断细胞有丝分裂，从而抑制肿瘤生长。它是临床治疗NSCLC的主要药物之一，单药有效率达到21%-24%，与顺铂联合有效率可达到42%^[1-3]^，是目前临床NSCLC的标准化疗方案之一。但普通紫杉醇难溶于水，目前国内外临床应用紫杉醇均是溶于聚氧乙基代与无水乙醇混合的复合溶媒中，而聚氧乙基代蓖麻油在体内降解时释放组胺导致严重的过敏反应与末梢神经毒性，这在很大程度上限制了紫杉醇的临床应用。

脂质体是一种由磷脂构成的类脂小球体，作为一种新型的药物载体具有降低药物毒性、改变药物的动力性质和组织分布、减少药物的消除速度、延长药物的作用时间等作用^[4]^。国外已有关于阿霉素脂质体、顺铂脂质体应用于临床的报道^[5]^。对于紫杉醇来说，脂质体主要是提高药物的水溶性，从而减轻毒副反应特别是变态反应的发生。

Straubinger等^[4]^报道紫杉醇脂质体静脉给药最大耐受量可达200 mg/kg，而普通紫杉醇最大耐受量仅30 mg/kg，且紫杉醇脂质体在动物体内毒性明显小于紫杉醇注射液。与普通紫杉醇相比，紫杉醇脂质体在肝、脾、肺等网状内皮系统较发达的脏器分布较多，并长时间保持在较高的浓度，在体内提供了一个缓慢释放药物的储存库，从而降低了药物的毒性^[6]^。目前国内已有多项临床研究^[7-11]^报道，在乳腺癌、NSCLC和小细胞肺癌等多种肿瘤的治疗中，紫杉醇脂质体和普通紫杉醇在用药剂量相同的情况下，临床近期疗效无明显差异。但在呼吸困难、面部潮红、皮疹、肌肉酸痛等方面，由于混合溶媒所造成的过敏反应，紫杉醇脂质体发生率明显低于普通紫杉醇。但目前国内关于紫杉醇脂质体是否能对晚期NSCLC患者远期生存带来获益的相关报道尚少，故本研究在观察紫杉醇脂质体与普通紫杉醇近期疗效的同时对所有患者进行了PFS和OS的统计观察，发现在相同剂量强度下LP组与TP组RR分别为26%和24%，两组比较无统计学差异，与国内外多数报道^[7-12]^一致。两组的PFS分别为5.1个月和4.2个月，OS分别为9.0个月和9.3个月，与目前多数晚期NSCLC一线治疗方案研究结果^[1-3, 10]^接近，且两组之间无统计学差异(*P*=0.110, *P*=0.342)。在血液学毒性、消化道反应、肝肾功能损害及脱发等方面两组发生率接近(*P* > 0.05)。但在肌肉酸痛、末梢神经毒性方面，LP组发生率明显低于TP组(8% *vs* 28%)，存在统计学差异(*P* < 0.05)，这也与之前国内多数报道结果相符^[7-11]^。分析原因可能为紫杉醇脂质体在体内的药代动力学发生改变，导致不良反应发生率降低^[6]^。但本研究中两组均未有过敏反应发生，考虑原因一方面可能与研究例数有限有关；另一方面紫杉醇作为一线抗肿瘤药物进入临床已经多年，临床医生对该药物的不良反应认识加深，临床预防用药经验不断丰富，加之药物生产厂家对药物工艺的不断改进，降低了过敏反应发生率。

本项临床研究结果虽然未能显示出在相同剂量强度下，紫杉醇脂质体相比普通紫杉醇未能给患者带来临床获益，但具有更高的安全性和临床耐受性，也从一定程度上预示紫杉醇脂质体可能具有更高的剂量强度应用范围。因此，能否通过提高剂量进而提高疗效，使患者更大程度获益值得临床进一步研究。
